# Interactions between the fovea and the periphery shape misbinding of visual features in a continuous report paradigm

**DOI:** 10.1038/s41598-024-78867-5

**Published:** 2024-11-17

**Authors:** Max Arwed Crayen, Stefan Treue, Moein Esghaei

**Affiliations:** 1 Cognitive Neuroscience Laboratory, German Primate Center - Leibniz Institute for Primate Research, Göttingen, Germany; 2 Faculty of Biology and Psychology, University of Göttingen, Göttingen, Germany; 3 International Max Planck Research School for Neurosciences, University of Göttingen, Göttingen, Germany; 4 School of Cognitive Sciences, Institute for Research in Fundamental Sciences (IPM), Tehran, Iran

**Keywords:** Perception, Sensory processing, Colour vision, Motion detection, Object vision

## Abstract

Human object perception depends on the proper integration of multiple visual features, such as color and motion. When features are integrated incorrectly, they are perceptually misbound and can cause illusions. This study investigates the phenomenon of continuous misbinding of color and motion features in peripheral vision, addressing the role of spatial continuity and color configuration in binding processes. Using a novel continuous report task, human subjects reported the perceived movement direction of color-coded dots in the peripheral visual field that were either congruent or incongruent with a foveal stimulus. Results indicate that spatial continuity is crucial for feature misbinding, with a marked decrease in peripheral perceptions bound to match foveal perception when the foveal and peripheral stimuli are spatially disjointed. Additionally, performance improved significantly when peripheral and foveal features were aligned, suggesting a ‘positive illusion’ effect where congruent features enhance perceptual accuracy. This effect was independent of the color pairings used, challenging the notion that color processing discrepancies might drive erroneous perception in this stimulus. These findings highlight the complex interplay between spatial configuration and perceptual accuracy in visual feature integration, with implications for understanding the neural basis of vision and developing applications to address perceptual inaccuracies in visual disorders.

**Supplementary Information:**

The online version contains supplementary material available at 10.1038/s41598-024-78867-5.

## Introduction

A visual scene is composed of many visual features, such as color, motion direction, shape, and orientation, that are perceptually joined into visual objects. This perceptual grouping of features is called feature binding^1^. The feature integration theory (FIT) hypothesizes nonspatial features, like color and shape, to be “free-floating” and unbound until spatial attention binds all features sharing the attended location into a perceptual object. Importantly, this process can fail, resulting in misbound features, i.e., illusory perceptions^2^. While misbindings have been described in various forms, feature misbindings have become popular prototypes for investigating binding processes^3–7^. Features are usually only bound when they are spatially close to each other but not when they are distant^8^. This binding of spatially close features is disturbed when top-down attentional processes are disrupted, as lesion studies showed that damages in parietal areas associated with top-down visual attention result in bindings of spatially distant features^9^. Spatial attention is thus hypothesized to determine the location from which non-spatial features that form an object are sampled^10^. Small attentional spotlights could, therefore, produce accurate feature bindings, while larger ones can cause more frequent binding errors.

Wu et al. introduced a stimulus that leads to the active binding of peripheral color and motion direction feature perception to align with foveal ones, resulting in a peripheral feature misbinding^11^. This stimulus design is remarkable as it allows a continuous misbinding experience over several seconds that alternates with a physically correct bound percept. In contrast, previous feature-misbinding stimuli often relied on short presentation times to create ambiguity^1,8,12,13^. It is unclear how this continuous bi-stability of correctly bound and misbound epochs is produced. It was hypothesized that this illusion would not only result from peripheral misbinding of features, but might also be due to a suboptimal processing of the colors red and green, starting in the retina^14^.

In this study, we explored the rules of this illusory stimulus to determine its suitability for investigating continuous feature-binding processes. Would the rate of illusory percepts change using different color pairings? This would reveal a dependency of this illusion on imperfect color processing in peripheral visual fields, as proposed by Gunther and McKinney^14^. Is poorer peripheral processing a crucial factor in the perception of this illusion? In that case, the spatial separation of foveal and peripheral stimulus parts should not influence the probability of illusory percepts. On the contrary, separating the peripheral stimulus spatially would make it easier to perceive it as a distinct object. As the two stimuli become increasingly distant, feature misbindings should become less frequent, thus indicating if this illusion results from peripheral misbinding processes over processing errors due to poor peripheral resolution.

## Methods

### Participants

Eighteen subjects (11 males, 21 to 30 years old) participated in this psychophysics study. All subjects had normal or corrected-to-normal vision. Each subject completed two experimental sessions and received written and oral instructions before the first session started. Before the first session, training was conducted to familiarize the subjects with the task and setup. First, they learned the task without any gaze restrictions. After calibrating the eye tracking system, they trained the task with gaze restriction to a central fixation point. This training was equal to the experimental task but discarded for analysis. After the training, the experiment started. This training was briefly repeated in the second experimental session before the experiment began. All subjects gave written informed consent before starting the first session. The procedures of the current study and the interaction with the participants, including the informed consent form, have been reviewed and approved by the Ethics Board of the Georg-Elias-Mueller-Institute of Psychology of the University of Göttingen. All methods were performed according to the relevant guidelines and regulations.

### Apparatus and stimuli

The stimuli were presented using MWorks 0.10 (MWorks-project.org) on a BenQ XL2720T monitor (resolution: 1920 × 1080 pixels, screen diagonal: 69 cm, refresh rate 60 Hz). During the experiments, the subjects’ heads were positioned at a viewing distance of 57 cm using a head and chin rest. This resulted in 32 pixels per degree of visual angle (dva). There was no additional light in the room. A central fixation point (diameter: 0.3 dva) was presented on the screen that the subjects had to foveate to start a trial. An eye tracker monitored eye movements at 1000 Hz (EYELINK 1000, SR Research, lens-eye distance: 55.5 cm), and trials were aborted if a subject’s gaze was further than five dva away from the center of the fixation cross. The participants used a joystick to report the perceived movement direction of the target random dot pattern (RDP). Starting from its neutral position, the joystick had to be moved beyond 30% of its maximum eccentricity for a response to be counted as valid. All responses above or below the horizontal meridian were considered upward or downward, respectively.

The stimuli used for this experiment were based on a steady-state misbinding stimulus first described by Wu et al.^11^. Two superimposed RDP groups were shown during each experimental trial (size: 36 × 18 dva^2^, dot diameter: 0.2 dva, dot speed: 3 dva/s, dot density: 5 dots/dva^2^). The dots of one group were moving up, and the ones of the other were moving down. Each group was divided into three sections. The central section (size: 18 × 18 dva^2^) was centered in the middle of the screen, and the left and right peripheral sections (sizes: 9 × 18 dva^2^ each) beyond nine dva of horizontal eccentricity to the left and right. Dots were either grey (RGB: 127, 127, 127), red (RBG: 215, 0, 0), green (RGB: 0, 142, 0), yellow (RGB: 70, 50, 0), or blue (RGB: 0, 48, 255). Each color’s luminance was measured (MINOLTA LS-10) and set to be equal at 15.8 cd/m^2. The background was black.

**Fig. 1:**
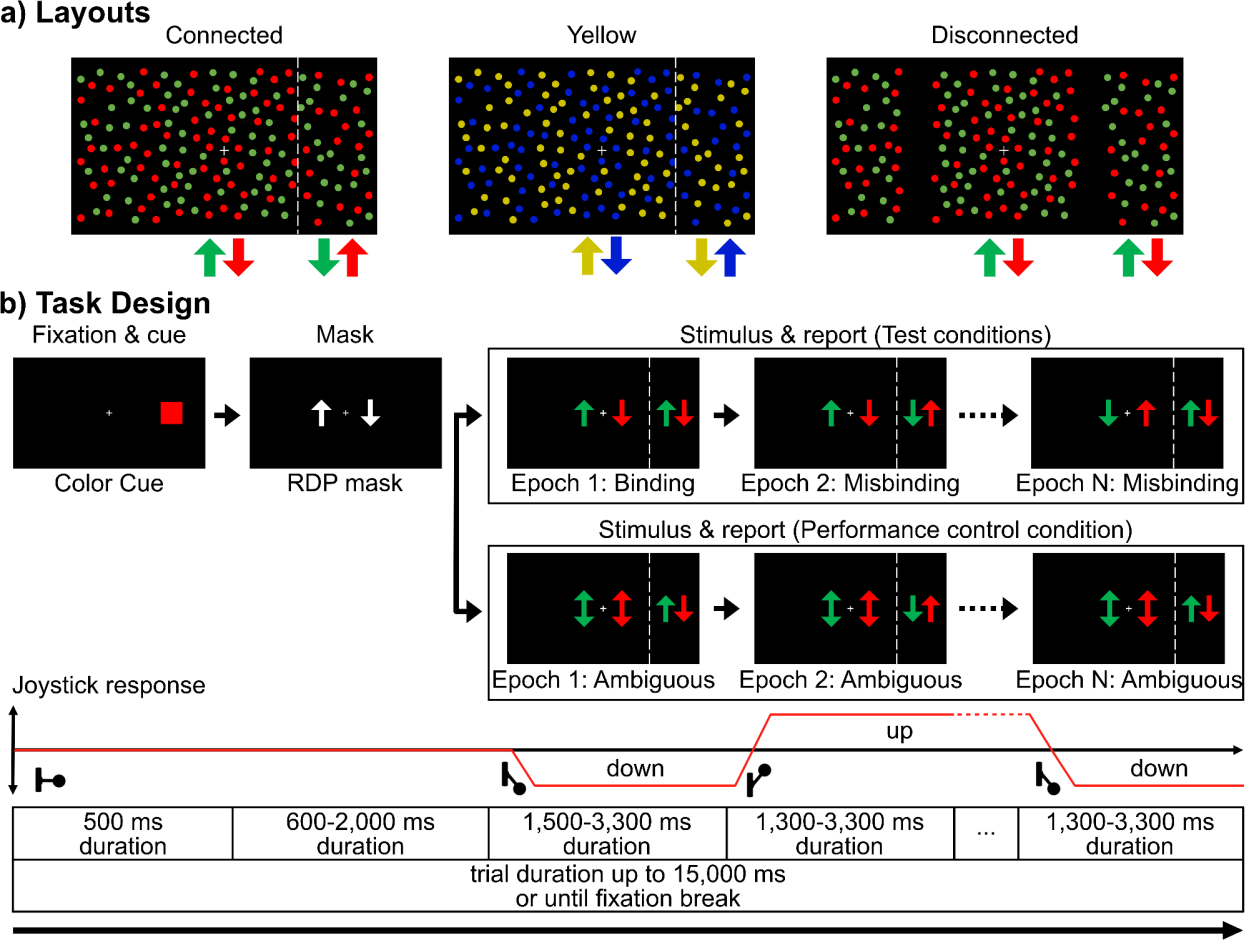
Task Design and stimulus layouts. (**a**) The three different stimulus layouts. The connected layout features red and green dots and a peripheral stimulus section that seamlessly connects to the central stimulus section. White dashed lines are displayed to visualize the border of central and peripheral stimuli, but they are not visible during the experiment. The yellow layout is identical to the connected layout but features yellow and blue instead of red and green dots. The disconnected layout again features red and green dots, but the peripheral stimulus section is disconnected from the central one by a small gap without dots. (**b**) Task design. In every trial, one layout is chosen randomly. The subject is cued for the target periphery (always right) and the target color of the dots they are supposed to report the movement direction of. After the cue disappears, an RDP mask appears. This mask consists of grey dots, with an equal number of dots moving up and down. Depending on the condition, the first stimulus displayed is either an ambiguous, binding, or misbinding stimulus. If it is an ambiguous stimulus, all successive epochs of this trial will also be ambiguous, but the direction of the peripheral target dots might change over time. After the first display of a binding or misbinding stimulus, the direction of the central or peripheral dots might change for the next epoch. This may lead to switches from binding to misbinding or misbinding to binding conditions. After up to 15 s, a trial ends, or when fixation is broken before that. The subjects are tasked to continuously indicate the movement direction of the cued peripheral dots with a joystick.

Three different stimulus layouts were used in this experiment (Fig. 1a). The first featured red and green dots with the peripheral sections placed seamlessly to connect with the central section (connected layout). The second was matching the first layout, but the color pairings were changed to yellow and blue (yellow layout). The third layout featured red and green dots, the central section had a diameter of 17 dva instead of 18, and the peripheral sections were placed 0.25 dva more eccentric on the horizontal meridian, generating a 0.75 dva wide blank gap between the central and peripheral sections of dots (disconnected layout).

### Task design

Subjects had to foveate the central fixation point and had to keep foveating it while reporting the movement direction of colored dots displayed in the right peripheral section of the stimulus (Fig. 1b). When starting a trial by moving the joystick briefly into any direction and then returning to the neutral position, one of the three possible stimulus layouts was chosen randomly. A red or yellow square was displayed for 500 ms on the right side of the screen to indicate the target’s color. After the cue’s disappearance, a masking period followed, which showed the target stimulus. However, all dots were grey, half moving upwards and the other half downwards. The mask was displayed for a random duration of 600 to 2000 ms. Then, the dots turned colorful. Half of the dots in each section turned to the target color, and the other half to their paired color. In the central section, dots could move in three different patterns. In the ambiguous condition’s pattern, half of the dots of each color group were moving upwards and the other half downwards. Alternatively, all central red dots moved upwards and green dots downwards, or greens moved upwards and reds downwards. The left peripheral section’s dot movement pattern always matched the central one’s. In the right peripheral section, all red dots were either moving upward and green dots downward, or green dots were moving upward and red dots downward. Stimulus configurations did not change during individual epochs of a trial and were grouped into three conditions. When peripheral dot movement directions matched the movement direction of same-colored central dots, these epochs were classified as binding epochs, when they were opposite as misbinding epochs. An epoch was considered ‘ambiguous’ if the central stimulus pattern was ambiguous, regardless of the peripheral target’s direction. The subjects had to continuously indicate their perceived movement direction of the cued dots by moving the joystick either up or down. Subjects did not receive feedback on whether their responses were correct during trials, and trials were not aborted if wrong responses were given. If the joystick remained in neutral position for more than 1300 ms, a trial was aborted. As long as continuous responses were given, a trial continued with possible direction changes after a randomly chosen period of 1500 to 3300 ms. Subjects had to complete 100 binding epochs, 100 misbinding epochs, and 33 ambiguous epochs in each of the three layouts, resulting in 699 epochs per session. After each epoch, a random stimulus configuration was chosen from possible combinations remaining for each condition. Random directions fitting the selected condition were chosen, which could result in a change of dot movement directions for the central and peripheral stimuli. A trial ended once a trial length of 15000 ms was reached or the foveation of the fixation point was disrupted. Epochs during which fixation breaks occurred were discarded and later repeated.

### Data analysis

To calculate the subjects’ performance during an epoch, we calculated the proportion of each epoch where the subject’s response direction equaled the physical stimulus direction. This proportion was corrected for the subject’s reaction time during that epoch. All epochs of one condition across all sessions of a subject were pooled for the statistical analysis. Trials aborted due to a fixation break or the subject not responding within 1300 ms after stimulus onset were excluded.

**Fig. 2:**
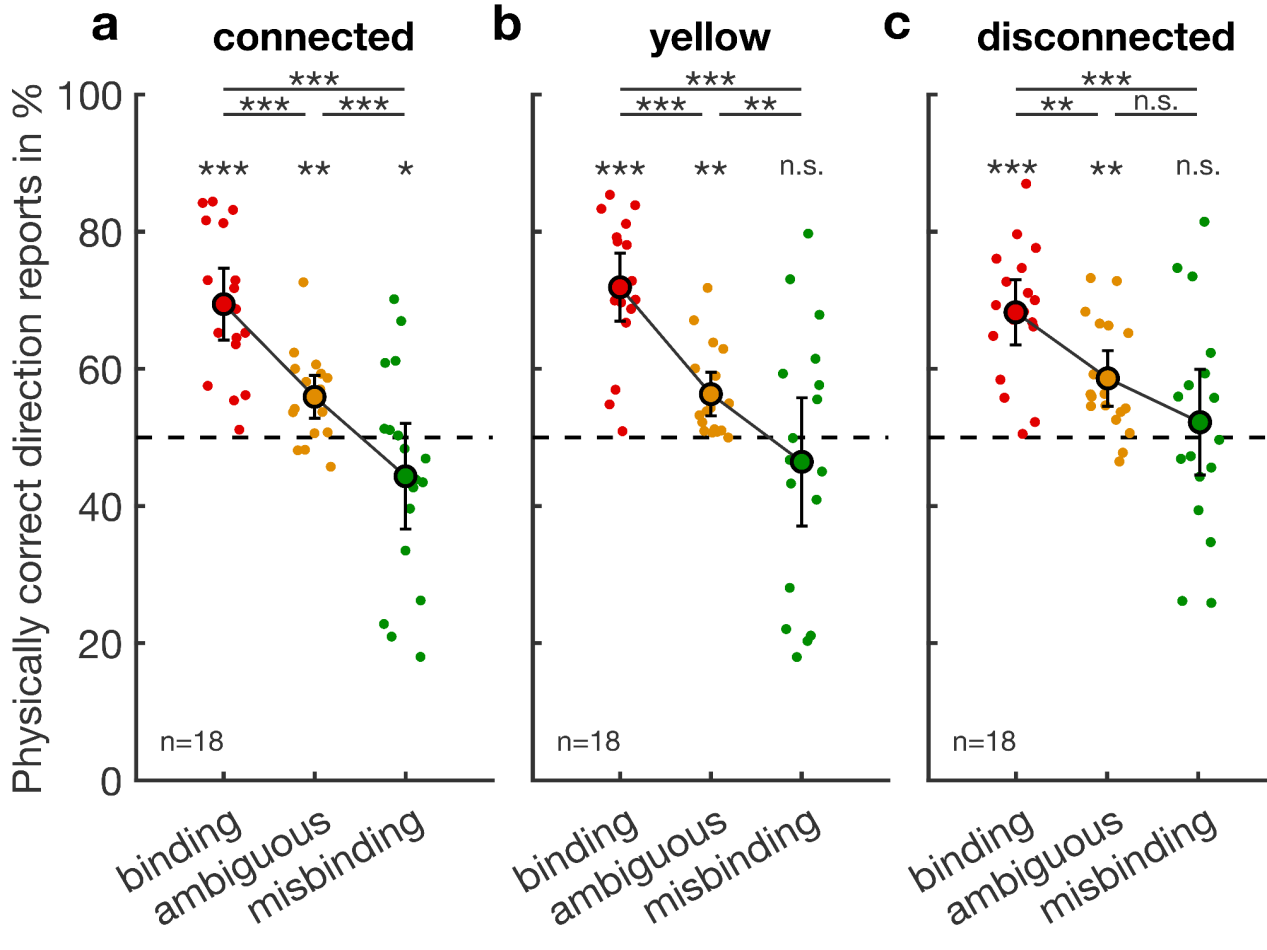
Task performance per condition in three different layouts. Each panel shows performance in binding, ambiguous, and misbinding conditions of one layout. (**a**) Task performance per condition in the connected layout. (**b**) Task performance per condition in the yellow layout. (**c**) Task performance per condition in the disconnected layout. Stars with lines indicate tests between the two groups connected by the lines. Stars without lines indicate tests against chance level (dashed line). *: *p* < 0.05, **: *p* < 0.01, ***: *p* < 0.001, *n. s.* not significant.

To test for the effects of binding and layout conditions (Fig. 2), we first averaged the performance separately per each combination of binding conditions, layout conditions, and subjects. The resulting average performance scores are theoretically bound within 0 and 1, and hence, we fitted a generalized linear mixed model (GLMM)^15^ with beta error distribution and logit-link function^16,17^ (Supplementary Table S1). In the fixed effects part of the model, we included binding condition, layout condition, and their interaction. In the random effects part, we included random intercept effects for the ID of the subjects and random slopes^18^ of binding condition and layout condition after manually dummy coding and then centering the two factors. We fitted the model using the function glmmTMB of the equally named package (version 1.1.9)^19^ in R (version 4.4.1)^20^. We determined confidence limits of model estimates and fitted values using parametric bootstraps (*n* = 1000 bootstraps; function *simulate* of the package glmmTMB). As an overall test of the effect of binding condition, layout condition, and their interaction, and to avoid ‘cryptic multiple testing’^21^, we compared the full model with a null model comprising only the intercept in the fixed effect part. We tested for the significance of the two-way interaction by dropping it from the model and comparing the fit of the resulting model with that of the full model. Both these tests utilized a likelihood ratio test^22^. The dataset used for the model comprised 162 performance scores for 18 individuals. The response was not overdispersed, given the model (dispersion parameter: 0.80). For pairwise post-hoc comparisons, we determined the differences in fitted values in two condition combinations of the design for each bootstrap. Then, we determined the proportions smaller than or equal to 0 and larger than or equal to 0 and multiplied the smaller of these two proportions by two. This value was taken as the two-tailed p-value for the difference between the two conditions. For this and all other models we determined model stability by dropping participants from the data set, one at a time, fitting the full model to each of the subsets, and finally comparing the range of estimates obtained for the subsets with those revealed for the full data set.

**Fig. 3:**
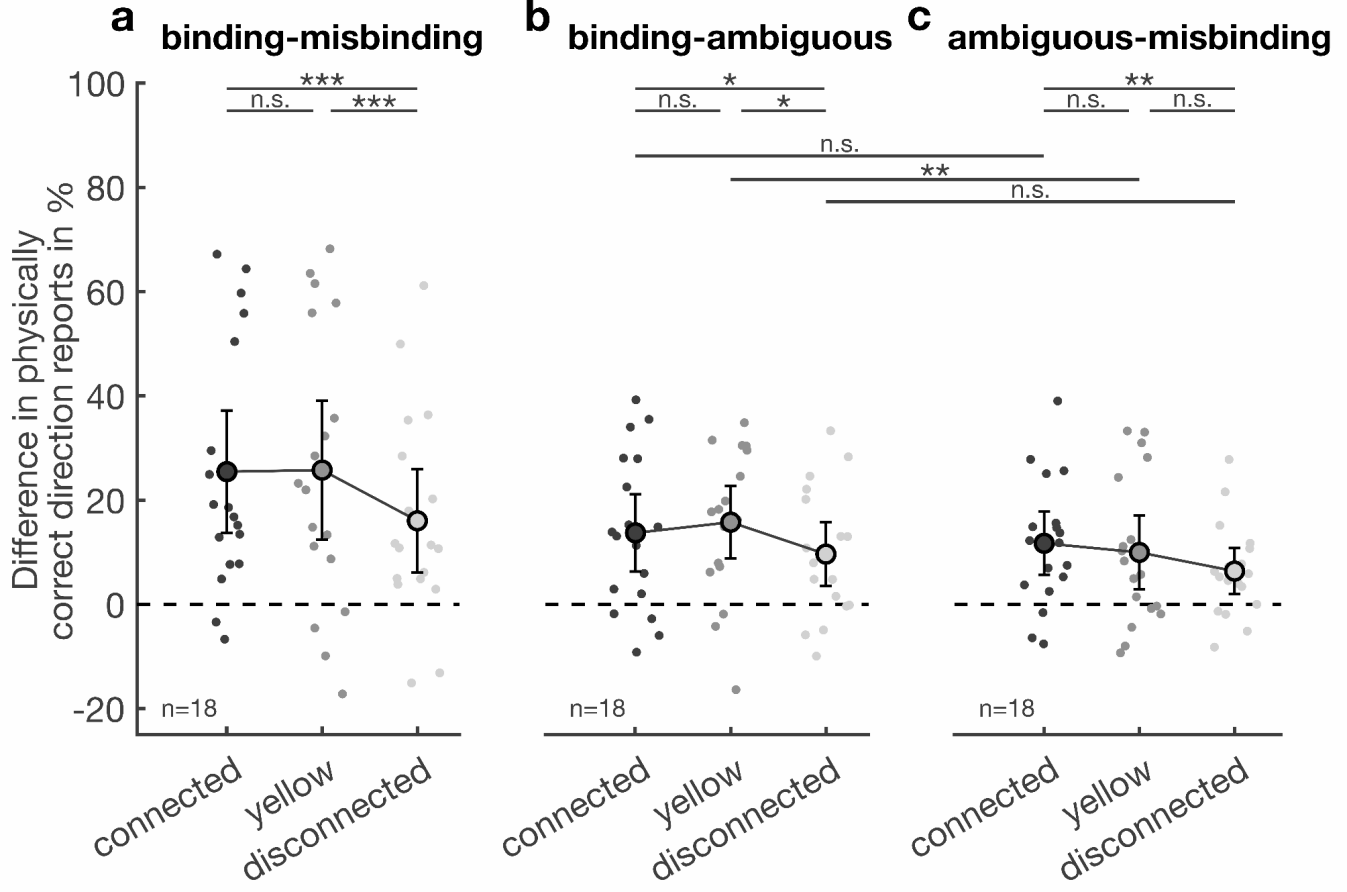
Active binding effect sizes per layout. The two conditions’ respective differences are shown for the connected, yellow, and disconnected layouts. (**a**) Performance differences of binding and misbinding conditions per layout. (**b**) Performance differences of binding and ambiguous conditions per layout. (**c**) Performance differences of ambiguous and misbinding conditions per layout. Stars with lines indicate tests between the two groups connected by the lines. *: *p* < 0.05, **: *p* < 0.01, ***: *p* < 0.001, *n. s.* not significant.

To test for the effects of different layouts on the performance difference between two binding conditions (Fig. 3), we again fitted a model with the same structure as described above (Supplementary Table S2). As the response was theoretically bound between − 100 and 100, respectively, we rescaled it to a theoretical range from 0 to 1 and fitted the model with a beta error structure and logit link function. In this model, the response was not overdispersed (dispersion parameter: 0.69). The dataset used for the model comprised 162 performance scores for 18 individuals.

The data were pre-processed using MATLAB 2023b (Mathworks Inc., Natnick, Massachusetts, USA) and plotted using Pierre Morel’s gram plotting library for MATLAB^23^.

## Results

To investigate how spatial and color relationships influence the perception of visual features, this study explores the roles of spatial continuity and different color pairings in a continuous feature misbinding task. Subjects continuously report the perceived movement direction of cued dots (target) in the peripheral panels of a transparent motion RDP stimulus, ignoring the foveated central panel’s movements (Fig. 1a). The peripheral dot movements either match (binding condition) or mismatch (misbinding condition) same-colored central dot movements. To determine ‘baseline’ performance, a control condition features colored central dots that do not carry directional information (ambiguous condition). Performance is computed as the correlation of the joystick’s response direction with the target stimulus’ physical movement direction. The statistical analysis was also performed using ANOVAs, assuming performance to be a continuous variable with homogeneous variance (Supplementary Methods). The results of both analyses show only minor differences.

### Active binding of peripheral features

To test whether the binding conditions and different layouts affect the performance, we compared the full performance model against a null model without the fixed effects part of the binding, layout condition, and their interaction. This test showed a significant effect by either the binding or layout condition or their interaction (χ^2^(8) = 58.54, *p* < 0.001). Afterward, we tested whether the binding and layout conditions were interacting with each other to justify pairwise comparisons and found a significant interaction between the binding and layout conditions (χ^2^(4) = 27.11, *p* < 0.001).

To ensure that the subjects could successfully report the peripheral direction in the absence of active binding in the connected layout (Fig. 2a), we determined whether subjects performed significantly above chance level in the ambiguous control condition. This was the case (Mean ± s.e.m. = 56%±1.5%, *p* < 0.01).

Next, we verified whether the binding (69%±2.5%) and misbinding (44%±3.7%) conditions induce active perceptual binding of peripheral features to align with foveal features, which would result in performances that were different from the baseline. Pairwise comparisons revealed significant differences between the ambiguous and binding conditions (*p* < 0.001) and the ambiguous and misbinding conditions (*p* < 0.001). The performance changed depending on the match or mismatch of peripheral and foveal feature pairings because performances in binding epochs were significantly different from misbinding ones (*p* < 0.001). This shows that the binding and misbinding conditions induce active binding of peripheral features foveal ones. During the binding condition, performance was elevated as foveal features matched peripheral ones, and during the misbinding condition, performance dropped due to the mismatch of foveal and peripheral features.

The yellow layout (Fig. 2b) used yellow and blue pairings instead of red and green to test the color dependency of active binding. We repeated the analysis for the yellow layout to validate whether the effect of active binding is also present in the binding (72%±2.3%) and misbinding (46%±4.4%) conditions of this layout. The performance in ambiguous epochs was significantly above baseline (56%±2.5%, *p* < 0.01), showing that accurately reporting peripheral features is possible. Pairwise comparisons showed significant differences between ambiguous and binding (*p* < 0.001), ambiguous and misbinding (*p* < 0.01), and binding and misbinding (*p* < 0.001) epochs. Thus, the yellow layout also induces perceptual misbinding of peripheral features, demonstrating the illusion’s independence from specific color combinations.

Next, we examined whether active binding occurs in binding (68%±2.2%) and misbinding (52%±3.6%) conditions when the peripheral and foveal stimulus parts are spatially disconnected by a blank gap (Fig. 2c). In ambiguous epochs where active binding was absent, performance was significantly above baseline (59%±1.9%, *p* < 0.001), demonstrating that the report of the feature perception in the peripheral stimulus is possible. Pairwise comparisons showed significant differences between ambiguous and binding (*p* < 0.01) and binding and misbinding (*p* < 0.001) epochs. The difference between ambiguous and misbinding epochs was not significant, but it showed the same trend as in the other conditions (*p* = 0.058). Hence, matching and mismatching foveal features induce active binding of peripheral features in the disconnected layout where the peripheral and foveal stimulus parts are spatially separated. This active binding effectively increases or reduces task performance due to illusory perceptions.

### The strength of active misbinding depends on the stimulus layout

To test whether the effect size of active binding of peripheral features would depend on different layouts, we compared the full model of the performance differences between binding conditions against a null model without the fixed effects part. This test showed a significant effect by either the binding or layout condition or their interaction (χ^2^(8) = 43.44, *p* < 0.001). Afterward, we tested whether the binding and layout conditions were interacting with each other to justify pairwise comparisons and found a significant interaction between the binding and layout conditions (χ^2^(4) = 11.70, *p* < 0.01).

Even though all layouts could induce misbinding, we evaluated if the strength of active binding changed depending on the stimulus layout. Any differences would be most pronounced when examining the difference between binding and misbinding epoch performances, as they show the most positive and negative effects, respectively, for the connected (mean difference ± s.e.m. = 25.2%±5.5%), yellow (25.5%±6.2%), and disconnected (15.9%±4.6%) layout (Fig. 3a). This effect of active binding was reduced in the disconnected layout as there were significant differences between the disconnected and the connected (−9.3%±1.8%, *p* < 0.001) and yellow (−9.6%±3.1, *p* < 0.001) layout. At the same time, there was no significant difference between the two different color layouts (*p* = 0.81). This suggests that active binding, though still present, is less likely to occur when peripheral features are spatially disconnected. Different colors do not affect this process, resulting in active binding effects of the same magnitude.

Further, we evaluated whether this reduced effect in the disconnected layout might be due to a reduction of active binding in only binding or misbinding epochs. This would result in a significant change in either the misbinding or the binding effect, i.e., the performance difference between the ambiguous and the misbinding or the binding condition. For the binding effect in connected (13.6%±3.5%), yellow (15.6%±3.2%), and disconnected (9.5%±2.9%) epochs, pairwise comparisons showed a significant difference between the disconnected and yellow layout (−6.1%±2.0%, *p* < 0.05) as well as the connected and yellow layout (−4.0%±1.7%, *p* < 0.05) (Fig. 3b). The misbinding effect, derived from the difference between ambiguous and misbinding epoch performance for each, the connected (11.6%±2.8%), yellow (9.9%±3.3%), and disconnected (6.3%±2.1%) layouts (Fig. 3c). Here, the pairwise comparisons showed a significant difference between the disconnected and connected layout (−5.3%±1.8%, *p* < 0.01). Together, these results suggest that disconnected peripheral stimuli similarly decrease the binding and misbinding of that stimulus’ features.

Notably, the performance during misbinding epochs was not significantly different from chance level performance, regardless of the used layout condition (Fig. 2a–c). Compared to the ambiguous condition, this reduction in performance could result from a misbinding effect that, by coincidence, reduces the performance in an amount that leads to a performance at 50% correct responses. If this is the case, this effect of active binding should be of the same magnitude in the opposite direction during binding epochs. Thus, we tested this hypothesis by comparing the active binding effect sizes for binding and misbinding conditions within layouts. Here, we observed a higher effect in the yellow layout’s binding condition rather than the misbinding condition (5.7%±2.1%, *p* < 0.01) (Fig. 3b,c). We did not observe notable differences in the active binding strengths in the two other layouts. The lower misbinding effect in misbinding epochs in the yellow layout might indicate that this condition does not exclusively reflect the impact of misbound perceptions. However, if the active binding process were impaired in this layout, we would expect a similarly diminished effect in the binding condition of the yellow layout as well. Since such an effect is absent and we do not observe similar effects in the other two layouts, we do not suspect that the slightly smaller misbinding effect reflects any processing differences in the yellow layout. Especially since we did not observe any differences in the overall binding effect of the connected and yellow layout (Fig. 3a), this supports the hypothesis that active binding occurs equally with matching and mismatching foveal feature values. Thus, the performance at chance level during misbinding epochs does not reflect subjects responding randomly. Rather, their perception is altered to perceive the target moving into each of the two directions equally often.

In summary, our data show that active binding is possible in a continuous report task featuring physical and illusory changes in the movement direction of colored peripheral dots. Furthermore, we could show that the baseline performance of this task can be measured by an additional condition that does not induce any active binding in the periphery. This illusory conjunction of color and motion features is not dependent on the colors used but showed a significant reduction when peripheral and foveal features were spatially separated instead of seamlessly connected.

## Discussion

Our study explored two critical aspects of perceptual misbinding in a novel continuous report task: the decrease in misbinding due to spatial discontinuity between central and peripheral stimuli and the enhancement of performance through a ‘positive illusion’ when features are congruent. Our findings confirm that spatial continuity plays a critical role in feature integration, with a marked decrease in misbinding observed when central and peripheral stimuli are spatially disjointed. Additionally, the observed improvement in performance, i.e., the rate of physically correct responses when peripheral and foveal features are aligned, is a previously neglected effect of active binding. Taken together, the results highlight a complex interplay between spatial configuration and perceptual accuracy in vision.

Many stimuli that induce illusory feature conjunction require short presentation times^1,3–8,12,13^, so it was an important adavance when Wu et al. introduced a stimulus that could induce steady–state misbinding of color and motion features in the peripheral field of view^11^. They found that the foveal perception of feature combinations would induce this misbinding of features in the periphery after ruling out that this illusion could result from mirroring feature information of the other periphery. Interestingly, the illusion-inducing, foveal part of the stimulus was spatially distant from the peripheral stimulus that was reported by subjects and perceived as misbound. The original design used a stimulus that did not physically change directions of motion over time. All reported direction changes were, therefore, due to perceptual misbinding.

However, it was quickly found that this illusory conjunction is not restricted to color and motion features. Studies based on color and orientation features showed that this illusion can be recreated with static images, only displayed for short periods of time^14,24^. Similarly, the illusory conjunction of color and motion features also does not rely on extended stimulus presentation times^25,26^. However, designs with short presentation times are less suited for fMRI, EEG, and single-cell electrophysiological neural recording techniques. In contrast, the long-lasting illusory perception of peripheral features being actively bound to align with foveal stimuli has already been extensively studied in human fMRI and EEG^27–29^. The most significant disadvantage of their design, which closely resembled the original task design introduced by Wu et al.^11^, was that it relied on the subject’s full cooperation in the task, as it was not easy to control whether they were reporting the stimuli as perceived. The movement directions of dots of one color did not change throughout one stimulus presentation. A subject had to understand and truthfully and attentively respond to the stimulus, while the experimenters had no way to control their performance. While this may be sufficient for human subjects, experiments with monkeys, often used in electrophysiological experiments, require some oversight and control to ensure the animals learn to report their perception rather than responding to their own (made-up) rules. Our new task design now introduces a task with rules that can, in principle, be learned by animals and humans while keeping the continuous aspect of the original design.

In our adapted paradigm, either the peripheral, the foveal, or both stimulus parts can inverse directions at randomized times, which forces subjects to report physical and illusory changes in the peripheral stimulus’ movement direction. By introducing another condition that features a foveal stimulus part that does not provide any directional information based on color groupings, we created a control condition that does not induce any perceptual active binding in the periphery. This design was inspired by Suzuki et al.^24^, who introduced a similar control condition in their experiment based on color and orientation feature conjunction. This allows for an effective control of baseline performances, as subjects need to to continuously pay attention to the stimulus, to ensure the perception of upcoming physical direction changes. This is also more effective than a control condition with an empty foveal section (tried in other designs^11,24,25^), as an empty center also reduces the perceptual load in the task, making the control task easier to respond to than the actual misbinding stimuli^24^. This paves the way for further electrophysiological experiments, as the perceptual load is constant in all stimuli, and the design allows long presentation times. This might help future experiments reveal the underlying neural mechanisms of this visual feature misbinding process. Thus, we expanded this illusory stimulus design’s usability by embedding it into a novel continuous task paradigm.

We observed that active binding of peripheral features is demonstrated not only by illusory misbinding, but also by an equally increasing performance during binding epochs. This suggests that the process active when perception is misbound can also improve performance when peripheral and foveal features match. While this was already visible from the original data acquired from Wu et al.^11^, it was impossible to properly quantify the strength of the illusion, as the blank center control condition did not qualify as a good baseline due to its dissimilar-shaped psychometric functions^24^. This ‘positive illusion’ was overlooked (but is noticeable in the published data) in previous studies. This happened as it was assumed that no active binding of peripheral features would happen when peripheral color and motion (or orientation) features already matched central ones^14,25,26,30^.

It is uncertain why illusory feature binding occurs, but it is hypothesized to be an ambiguity-solving mechanism. Illusory feature perceptions are more likely if the feature similarity is high and the spatial distance between these features is low^5,8^. Many designs that create illusory feature bindings rely on short presentation times to create ambiguity^1,8,12,13^, which is not the case here. Instead, the feature similarity between the behaviorally irrelevant central and relevant periphery is very high. This, and their spatial proximity, as they are seamlessly connected, might allow a perceptual generalization, causing the subject to illusorily perceive the stimulus as uniform despite the physical differences of feature combinations at different locations.

Such uniformity illusions are not always related to feature binding. Sometimes, they result from reconstructing the peripheral perception using more detailed information from the fovea, where photoreceptor density is much higher^31^. However, uniformity illusions only occur over time and remain steady once they occur, assuming the visual input does not change. The illusion examined in our experiment generates bi-stable perceptions, where perception switches between correct (physical) and illusory binding of features^28^. This suggests that this illusion results from feature-binding processes that misbind or correctly bind the different feature combinations over time. This could result from a probabilistic sampling of non-spatial features across the spatial extent of the attended object^10^. If the borders of an object of interest are spatially uncertain, a larger sampling area could lead to overweighting of incorrect feature combinations of adjacent areas, especially if the similarity of the behaviorally irrelevant features to that of target features is high^5^. Another facilitating factor could be the known processing of peripheral signals in visual cortex areas with foveal receptive fields^32^. This process enhances object recognition as the peripheral information processed in the retinotopically foveal regions is fed back to aid peripheral perception^33^. Information about foveal objects processed simultaneously may overwrite the peripheral information in this feedback loop, especially if they are presented simultaneously and comprise similar feature combinations.

We saw that active misbinding is efficiently reduced when the periphery and center are spatially disconnected. This aligns with previous findings that showed a similar effect for misbound color and motion feature conjunctions in the fovea based on perceptually dominant peripheral feature combinations^25^. The spatial disconnection might reduce the probability of assuming that the two stimulus parts are part of the same object, thus refining the feature sampling area to cover only the periphery, reducing the probability of active binding to match foveal features. It has been hypothesized that this illusion is not the result of a high-level process like active visual feature binding but rather an effect observable due to the used color scheme and how different colors are processed, in part already on the level of the retina^14^. Our results contradict this hypothesis and demonstrate that the color pairing does not influence the probability of active binding, matching other observations^26^. This suggests that the misbinding of features in this illusion is likely not due to poor separation of the processing of red and green colors. Instead, such feature misbinding occurs independently of the combinations used and the processing of different colors. This supports the idea that feature misbinding is a high-level process independent of unique processing characteristics of feature dimensions like color. A decrease in active binding is observed only when peripheral and foveal colors are increasingly dissimilar^30^. But this is just another example of feature dissimilarity and further supports the idea that active binding depends on feature similarity and less on the use of specific colors.

Although we observed baseline performances significantly above chance-level responses, the performance in the control condition was lower than in other studies that showed either random movement^24^ or blank stimulus parts to generate a control condition^11,25^. This might, in part, be because the border that separates central and peripheral dots was more eccentric in this experiment than in other experiments with similar layouts^11,14,24,30^. Other experiments had peripheral borders with similar eccentricities^27–29^. Experiments with similarly eccentric target stimuli did not report a control condition for assessing a subject’s baseline performances, so comparing the reported performances is impossible. Misbinding of peripheral features is more likely the more peripheral they are, as feature binding is increasingly impaired in peripheral vision^34^. One can apply an eccentricity-dependent cortical magnification factor to ensure that this is not just due to decreasing photo-receptor density in the peripheral field of view and less cortical area that processes this information^26^. This increases the relative size of peripheral stimuli to make them equally discriminable as foveal stimuli of the original size. This correction increases peripheral performance, but misbinding of peripheral features is still observed when subjects face a misbinding-inducing stimulus^26^. This persistence suggests a fundamental foveal-peripheral dichotomy in visual processing, resulting in a stronger representation of foveal over peripheral features^35,36^. As such, a proper misbinding stimulus design is a compromise between maximizing the misbinding probability by increasing the stimulus’ eccentricity while keeping it close enough for the retina and cortex to recognize the peripheral features in principle. Future studies could thoroughly investigate the ideal distance for peripheral stimuli to maximize the probability of misbinding of peripheral features. Furthermore, the continuous task design might be more demanding than a layout featuring short presentation times, which might negatively influence performance in baseline control conditions.

Studying misbinding is crucial when studying the binding problem^37–39^. Here, we provide support for the hypothesis that the active misbinding of peripheral features to central ones is an ambiguity-solving mechanism that reduces feature variations within one perceptual object. This is shown as the active binding probability decreases with increasing spatial separation and with increasing feature-dissimilarity in the peripheral and foveal parts^30^. Our novel continuous task design provides performance control while allowing the recording of active feature misbinding in continuous settings, which is particularly useful for electrophysiological studies in humans and animals.

## Electronic supplementary material

Below is the link to the electronic supplementary material.

Supplementary Material 1

## Data Availability

The data generated and analyzed during the current study are publicly available in the Göttingen Research Online (GRO) repository, https://data.goettingen-research-online.de/dataverse/ContMisbind.

## References

[1] Treisman, A. M. & Gelade, G. A feature-integration theory of attention. *Cogn. Psychol.* **12**, 97–136 (1980). 7351125 10.1016/0010-0285(80)90005-5

[2] Treisman, A. M. The binding problem. *Curr. Opin. Neurobiol.* **6**, 171–178 (1996). 8725958 10.1016/s0959-4388(96)80070-5

[3] Treisman, A. M. & Schmidt, H. Illusory conjunctions in the perception of objects. *Cogn. Psychol.* **14**, 107–141 (1982). 7053925 10.1016/0010-0285(82)90006-8

[4] Cohen, A. & Ivry, R. Illusory conjunctions inside and outside the focus of attention. *J. Exp. Psychol. Hum. Percept. Perform.* **15**, 650–663 (1989). 2531202 10.1037//0096-1523.15.4.650

[5] Ivry, R. B. & Prinzmetal, W. Effect of feature similarity on illusory conjunctions. *Percept. Psychophys.* **49**, 105–116 (1991). 2017349 10.3758/bf03205032

[6] Keele, S. W., Cohen, A., Ivry, R., Liotti, M. & Yee, P. Tests of a temporal theory of attentional binding. *J. Exp. Psychol. Hum. Percept. Perform.* **14**, 444 (1988). 2971772 10.1037//0096-1523.14.3.444

[7] Prinzmetal, W., Presti, D. E. & Posner, M. I. Does attention affect visual feature integration? *J. Exp. Psychol. Hum. Percept. Perform.* **12**, 361 (1986). 2943864 10.1037//0096-1523.12.3.361

[8] Prinzmetal, W., Henderson, D. & Ivry, R. Loosening the constraints on illusory conjunctions: assessing the roles of exposure duration and attention. *J. Exp. Psychol. Hum. Percept. Perform.* **21**, 1362–1375 (1995). 7490585 10.1037//0096-1523.21.6.1362

[9] Friedman-Hill, S. R., Robertson, L. C., Desimone, R. & Ungerleider, L. G. Posterior parietal cortex and the filtering of distractors. *Proc. Natl. Acad. Sci.* **100**, 4263–4268 (2003). 12646699 10.1073/pnas.0730772100 PMC153081

[10] Vul, E. & Rich, A. N. Independent sampling of features enables conscious perception of bound objects. *Psychol. Sci.* **21**, 1168–1175 (2010). 20631320 10.1177/0956797610377341

[11] Wu, D-A., Kanai, R. & Shimojo, S. Steady-state misbinding of colour and motion. *Nature* **429**, 262–262 (2004). 15152242 10.1038/429262a

[12] Moutoussis, K. & Zeki, S. A direct demonstration of perceptual asynchrony in vision. *Proc. R Soc. Lond. B Biol. Sci.* **264**, 393–399 (1997). 10.1098/rspb.1997.0056 PMC1688275 9107055

[13] Nishida, S. & Johnston, A. Marker correspondence, not processing latency, determines temporal binding of visual attributes. *Curr. Biol.* **12**, 359–368 (2002). 11882286 10.1016/s0960-9822(02)00698-x

[14] Gunther, K. L. & McKinney, M. R. Poor peripheral binding depends in part on stimulus color. *Atten. Percept. Psychophys.* **82**, 3606–3617 (2020). 32691368 10.3758/s13414-020-02086-z

[15] Baayen, R. H. *Analyzing Linguistic Data: A Practical Introduction to Statistics Using R* (Cambridge University Press, 2008).

[16] McCullagh, P. & Nelder, J. A. Generalized Linear Models, 2nd ed. 10.1201/9780203753736 (2019).

[17] Bolker, B. M. Ecological Models and Data in R. 10.1515/9781400840908 (2008).

[18] Barr, D. J., Levy, R., Scheepers, C. & Tily, H. J. Random effects structure for confirmatory hypothesis testing: keep it maximal. *J. Mem. Lang.* **68**, 255–278 (2013). 10.1016/j.jml.2012.11.001 PMC3881361 24403724

[19] Brooks, M. E. et al. glmmTMB balances speed and flexibility among packages for zero-inflated generalized linear mixed modeling. *R J.* **9**, 378–400 (2017).

[20] R Core Team. *R: A Language and Environment for Statistical Computing* (R Found. Stat. Comput, 2020).

[21] Forstmeier, W. & Schielzeth, H. Cryptic multiple hypotheses testing in linear models: overestimated effect sizes and the winner’s curse. *Behav. Ecol. Sociobiol.* **65**, 47–55 (2011). 21297852 10.1007/s00265-010-1038-5 PMC3015194

[22] Dobson, A. J. & Barnett, A. G. An Introduction to Generalized Linear Models, 4th ed. 10.1201/9781315182780 (2018).

[23] Morel, P. Gramm: grammar of graphics plotting in Matlab (2.24). 10.5281/zenodo.1188423 (2018).

[24] Suzuki, M., Wolfe, J. M., Horowitz, T. S. & Noguchi, Y. Apparent color–orientation bindings in the periphery can be influenced by feature binding in central vision. *Vis. Res.* **82**, 58–65 (2013). 23454501 10.1016/j.visres.2013.02.011

[25] Noguchi, Y., Shimojo, S., Kakigi, R. & Hoshiyama, M. An integration of color and motion information in visual scene analyses. *Psychol. Sci.* **22**, 153–158 (2011). 21177514 10.1177/0956797610393743

[26] Bi, K., Zhang, Y. & Zhang, Y-Y. Central-peripheral dichotomy: color-motion and luminance-motion binding show stronger top-down feedback in central vision. *Atten. Percept. Psychophys.* **84**, 861–877 (2022). 35304697 10.3758/s13414-022-02465-8

[27] Zhang, X., Qiu, J., Zhang, Y., Han, S. & Fang, F. Misbinding of color and motion in Human visual cortex. *Curr. Biol.* **24**, 1354–1360 (2014). 24856212 10.1016/j.cub.2014.04.045

[28] Zhang, Y., Zhang, Y., Cai, P., Luo, H. & Fang, F. The causal role of α-oscillations in feature binding. *Proc. Natl. Acad. Sci.* **116**, 17023–17028 (2019). 31383766 10.1073/pnas.1904160116 PMC6708338

[29] Zhang, Y., Zhang, X., Wang, Y. & Fang, F. Misbinding of color and motion in human early visual cortex: evidence from event-related potentials. *Vis. Res.* **122**, 51–59 (2016). 27038562 10.1016/j.visres.2015.12.010

[30] Wang, W. & Shevell, S. K. Do S cones contribute to color-motion feature binding? *J. Opt. Soc. Am. A* **31**, A60 (2014). 10.1364/JOSAA.31.000A60 24695203

[31] Otten, M., Pinto, Y., Paffen, C. L. E., Seth, A. K. & Kanai, R. The uniformity illusion: Central stimuli can determine peripheral perception. *Psychol. Sci.*10.1177/0956797616672270 (2016). 28078975 10.1177/0956797616672270

[32] Stewart, E. E. M., Valsecchi, M. & Schütz, A. C. A review of interactions between peripheral and foveal vision. *J. Vis.* **20**, 2–2 (2020). 33141171 10.1167/jov.20.12.2 PMC7645222

[33] Williams, M. A. et al. Feedback of visual object information to foveal retinotopic cortex. *Nat. Neurosci.* **11**, 1439–1445 (2008). 18978780 10.1038/nn.2218 PMC2789292

[34] Neri, P. & Levi, D. M. Spatial resolution for feature binding is impaired in peripheral and amblyopic vision. *J. Neurophysiol.*10.1152/jn.01261.2005 (2006). 16421195 10.1152/jn.01261.2005

[35] Zhaoping, L. A new framework for understanding vision from the perspective of the primary visual cortex. *Curr. Opin. Neurobiol.* **58**, 1–10 (2019). 31271931 10.1016/j.conb.2019.06.001

[36] Xue, S., Fernández, A. & Carrasco, M. Featural representation and internal noise underlie the eccentricity effect in contrast sensitivity. *J. Neurosci.*10.1523/JNEUROSCI.0743-23.2023 (2024). 38050093 10.1523/JNEUROSCI.0743-23.2023 PMC10860475

[37] Seymour, K., Clifford, C. W. G., Logothetis, N. K. & Bartels, A. The coding of color, motion, and their conjunction in the human visual cortex. *Curr. Biol.* **19**, 177–183 (2009). 19185496 10.1016/j.cub.2008.12.050

[38] Whitney, D. Neuroscience: toward unbinding the binding problem. *Curr. Biol.* **19**, R251–R253 (2009). 19321141 10.1016/j.cub.2009.01.047 PMC2857400

[39] Zhang, Y., Zhang, Y-Y. & Fang, F. Neural mechanisms of feature binding. *Sci. China Life Sci.* **63**, 926–928 (2020). 32133593 10.1007/s11427-019-1615-4

