# Supplementary material for: Interactions between the fovea and the periphery shape misbinding of visual features in a continuous report paradigm
Source: Sci Rep. 2024 Nov 17;14:28381. doi: 10.1038/s41598-024-78867-5 (PMC11570667; doi:10.1038/s41598-024-78867-5)
Supplement: Supplementary file 1 — Supplementary Material 1 [file 41598_2024_78867_MOESM1_ESM.pdf]

Supplementary Material

“Interaction between the fovea and the periphery shape misbinding of visual features in a continuous report paradigm”

Supplementary Table S1. *Results of the generalized linear mixed model fitted to the performance values of each subject, binding condition, and layout condition combination.*

	Estimate	Std. Error	X2.5	X97.5	min	max
(Intercept)	0,243	0,094	0,0648	0,4406	0,2003	0,2673
Binding	0,616	0,15	0,3171	0,9209	0,5465	0,6766
Misbinding	-0,495	0,126	-0,7535	-0,2569	-0,5436	-0,4216
Disconnected	0,109	0,062	-0,0154	0,2318	0,0763	0,1331
Yellow	0,015	0,06	-0,0933	0,1353	-0,0026	0,0339
Binding:Disconnected	-0,175	0,089	-0,3576	0,0027	-0,2052	-0,1440
Misbinding:Disconnected	0,238	0,087	0,0769	0,4101	0,2015	0,2814
Binding:Yellow	0,107	0,089	-0,0682	0,2963	0,0403	0,1532
Misbinding:Yellow	0,075	0,086	-0,0880	0,2404	0,0488	0,1031

Supplementary Table S2. *Results of the generalized linear mixed model fitted to the performance difference values of each subject, binding condition, and layout condition combination.*

	Estimate	Std. Error	X2.5.	X97.5.	min	max
(Intercept)	0,237	0,082	0,0704	0,3978	0,2037	0,2603
Binding-Ambiguous	0,04	0,044	-0,0435	0,1273	0,0157	0,0664
Binding-Misbinding	0,311	0,073	0,1730	0,4589	0,2732	0,3403
Disconnected	-0,109	0,039	-0,1833	-0,0346	-0,1289	-0,0913
Yellow	-0,035	0,044	-0,1198	0,0498	-0,0483	-0,0224
Binding-Ambiguous:Disconnected	0,025	0,048	-0,0721	0,1175	0,0060	0,0581
Binding-Misbinding:Disconnected	-0,099	0,048	-0,2005	-0,0046	-0,1156	-0,0840
Binding-Ambiguous:Yellow	0,078	0,048	-0,0146	0,1770	0,0505	0,1018
Binding-Misbinding:Yellow	0,046	0,049	-0,0515	0,1407	0,0159	0,0675

Supplementary Methods - Statistical analysis using ANOVA

To calculate the subjects' performance during an epoch, we calculated the time proportion of each epoch where the stimulus direction and the subject's response direction were the same. This proportion was corrected for the subject's reaction time during that epoch. All epochs of one condition across all sessions of a subject were pooled for the statistical analysis. Trials aborted due to a fixation break or the subject not responding within 1300 ms after stimulus onset were excluded.
Subject performances for each condition were z-scored and tested for standard normal distribution using a Kolmogorow-Smirnow-Test before comparing them against each other. All groups were normally distributed. Repeated measures ANOVAs were performed to test for significant differences between conditions within layouts. Paired t-tests were used to compare the two groups in post-hoc tests. Post-hoc tests were corrected using the Holm-Bonferroni method to counteract the problem of multiple comparisons. The data were analyzed using MATLAB 2023b (Mathworks Inc., Natnick, Massachusetts, USA) and plotted using Pierre Morel's gram plotting library for MATLAB [17].

<u>Results</u>

To investigate how spatial and color relationships influence the perception of visual features, this study explores the roles of spatial continuity and different color pairings in a continuous feature misbinding task. Subjects continuously report the perceived movement direction of cued dots (target) in the peripheral panels of a transparent motion RDP stimulus, ignoring the foveated central panel's movements (Fig 1a). The peripheral dot movements either match (binding condition) or mismatch (misbinding condition) same-colored central dot movements. To determine 'baseline' performance, a control condition features colored central dots that do not carry directional information (ambiguous condition). Performance is computed as the correlation of the joystick's response direction with the target stimulus' physical movement direction.

<u>Active binding of peripheral features</u>

To ensure that the subjects could successfully report the peripheral direction in the absence of active binding in the connected layout (Fig 2a), we determined whether subjects performed significantly above chance level in the ambiguous control condition. This was the case (Mean ± s.e.m. = 56%±1.5%, $t(17) = 4.0$, $p < 0.001$).
Next, we verified whether the binding and misbinding conditions induce active perceptual binding of peripheral features to align with foveal features, which would result in performances that were different from the baseline. The task performance changed significantly depending on the color and motion direction pairing of the foveal stimulus as we observed significant differences between the performances in the binding (69%±2.5%), misbinding (44%±3.7%), and ambiguous conditions (rANOVA $F(2,34) = 18.9$, $p < 0.001$). Post-hoc tests revealed significant differences between the ambiguous and binding conditions ($t(17) = -3.9$, $p<0.01$) and the ambiguous and misbinding conditions ($t(17) = -4.1$, $p<0.001$). The performance changed depending on the match or mismatch of peripheral and foveal feature pairings because performances in binding trials were significantly different from misbinding ones ($t(17) = -4.6$, $p<0.001$). This shows that the binding and misbinding conditions induce active binding of peripheral features foveal ones. During the binding condition, performance was elevated as

foveal features matched peripheral ones, and during the misbinding condition, performance dropped due to the mismatch of foveal and peripheral features.

The yellow layout (Fig 2b) used yellow and blue pairings instead of red and green to test the color dependency of active binding. We repeated the analysis for the yellow layout to validate whether the effect of active binding is also present in this layout. The performance in ambiguous trials was significantly above baseline (56%±1.5%, $t(17) = 4.2$, $p<0.001$), showing that accurately reporting peripheral features is possible. As in the connected layout, we observed significant differences between the performances in ambiguous, binding (72%±2.3%), and misbinding (46%±4.4%) trials ($F(2,34) = 16.4$, p<001), indicating that foveal feature conjunctions influence the perception of the periphery. Post-hoc tests showed significant differences between ambiguous and binding ($t(17) = -4.8$, $p<0.001$), ambiguous and misbinding ($t(17) = -3.0$, $p< 0.01$), and binding and misbinding ($t(17) = -4.1$, $p<0.001$) trials. Thus, the yellow layout also induces perceptual misbinding of peripheral features, demonstrating the illusion's independence from specific color combinations.

Next, we examined whether active binding occurs when the peripheral and foveal stimulus parts are spatially disconnected by a blank gap (Fig 2c). In ambiguous trials where active binding was absent, performance was significantly above baseline (59%±1.9%, $t(17) = 4.5$, $p<0.001$), demonstrating that the report of the feature perception in the peripheral stimulus is possible. The match of mismatch of peripheral and foveal features resulted in significant differences in the performances in ambiguous, binding (68%±2.2%), and misbinding (52%±3.6%) trials ($F(2,34) = 11.2$, p<001). Post-hoc tests showed significant differences between ambiguous and binding ($t(17) = -3.3$, $p<0.01$), ambiguous and misbinding ($t(17) = -3.0$, $p< 0.01$), and binding and misbinding ($t(17) = 3.4$, $p<0.01$) trials. Hence, matching and mismatching foveal features induce active binding of peripheral features in the disconnected layout where the peripheral and foveal stimulus parts are spatially separated. This active binding effectively increases or reduces task performance due to illusory perceptions.

<u>The strength of active misbinding depends on the stimulus layout</u>

Even though all layouts could induce misbinding, we evaluated if the strength of active binding changed depending on the stimulus layout. Any differences would be most pronounced when examining the difference between binding and misbinding trial performances, as they show the most positive and negative effects, respectively. We observed that the performance difference between binding and misbinding performances was significantly different in connected (mean difference ± s.e.m. = 25.2%±5.5%), yellow (25.5%±6.2%), and disconnected layouts (15.9%±4.6%) ($F(2,34) = 9.7$, $p<0.001$) (Fig 3a). The effect of active binding was reduced in the disconnected layout as there were significant differences between the disconnected and the connected (-9.3%±1.8%, $t(17) = -7.6$, $p<0.001$) and yellow (-9.6%±3.1, $t(17) = -3.1$, $p<0.01$) layout. At the same time, there was no significant difference between the two different color layouts. This suggests that active binding, though still present, is less likely to occur when peripheral features are spatially disconnected. Different colors do not affect this process, resulting in active binding effects of the same magnitude.

Further, we evaluated whether this reduced effect in the disconnected layout might be due to a reduction of active binding in only binding or misbinding trials. This would result in a significant change in either the misbinding or the binding effect, i.e., the performance difference between the ambiguous and the misbinding or the binding condition. The binding effect in connected (13.6%±3.5%), yellow (15.6%±3.2%), and disconnected (9.5%±2.9%) trials was significantly different ($F(2,34) = 4.9$, $p<0.05$)

(Fig 3b). Post-hoc t-tests only showed a significant difference between the disconnected and yellow layout (-6.1%±2.0%, $t(17) = -3.0$, $p<0.01$). The misbinding effect, derived from the difference between ambiguous and misbinding trial performance, also showed significant differences for connected (11.6%±2.8%), yellow (9.9%±3.3%), and disconnected (6.3%±2.1%) layouts ($F(2,34) = 4.2$, $p<0.05$) (Fig 3c). Here, the post-hoc t-test showed a significant difference between the disconnected and connected layout (-5.3%±1.8%, $t(17) = -3.0$, $p<0.01$). Together, these results suggest that disconnected peripheral stimuli similarly decrease the binding and misbinding of that stimulus' features.

Notably, the performance during misbinding epochs was not significantly different from chance level performance, regardless of the used layout condition (Fig 2a-c). Compared to the ambiguous condition, this reduction in performance could result from a misbinding effect that, by coincidence, reduces the performance in an amount that leads to a performance at 50% correct responses. If this is the case, this effect of active binding should be of the same magnitude in the opposite direction during binding epochs. Thus, we tested this hypothesis by comparing the active binding effect sizes for binding and misbinding conditions within layouts. Here, we observed a moderately higher effect in the yellow layout's binding condition rather than the misbinding condition (5.7%±2.1%, $t(17) = -2.7$, $p<0.05$). We did not observe notable differences in the active binding strengths in the two other layouts. The lower misbinding effect in misbinding epochs in the yellow layout might indicate that this condition does not exclusively reflect the impact of misbound perceptions. However, if the active binding process were impaired in this layout, we would expect a similarly diminished effect in the binding condition of the yellow layout as well. Since such an effect is absent and we do not observe similar effects in the other two layouts, we do not suspect that the slightly smaller misbinding effect reflects any processing differences in the yellow layout. Especially since we did not observe any differences in the overall binding effect of the connected and yellow layout (Fig 3a), this supports the hypothesis that active binding occurs equally with matching and mismatching foveal feature values. Thus, the performance at chance level during misbinding epochs does not reflect subjects responding randomly. Rather, their perception is altered to perceive the target moving into each of the two directions equally often.

In summary, our data show that active binding is possible in a continuous report task featuring physical and illusory changes in the movement direction of colored peripheral dots. Furthermore, we could show that the baseline performance of this task can be measured by an additional condition that does not induce any active binding in the periphery. This illusory conjunction of color and motion features is not dependent on the colors used but showed a significant reduction when peripheral and foveal features were spatially separated instead of seamlessly connected.
